# Fatal myocardial infarction associated with intravenous N-acetylcysteine error

**DOI:** 10.1186/1865-1380-4-54

**Published:** 2011-08-30

**Authors:** Andrew R Elms, Kelly P Owen, Timothy E Albertson, Mark E Sutter

**Affiliations:** 1 University of California, Davis, Department of Emergency Medicine, Medical Toxicology, PSSB 2100, 2315 Stockton Blvd, Sacramento, CA 95817 USA; 2 University of California, Davis, Department of Internal Medicine, Division of Pulmonary and Critical Care, Medical Toxicology, PSSB 3100, 4150 V Street, Sacramento, CA 95817 USA

**Keywords:** N-acetylcysteine, Myocardial Infarction, Formulation Error, Dosing Error, Anaphylactoid reaction

## Abstract

**Background:**

N-acetylcysteine is used to treat acetaminophen toxicity and is available in both intravenous and oral formulations. Our report describes a patient treated with intravenous N-acetylcysteine for acetaminophen toxicity who died after an anaphylactoid reaction following initiation of the infusion.

**Objective:**

Clinicians should be aware of potential complications when deciding on which formulation of N-acetylcysteine to administer.

**Case Report:**

A 53-year-old male presented with altered mental status after an overdose of acetaminophen/hydrocodone and carisoprodol. He had an acetaminophen level of 49 mcg/ml with an unknown time of ingestion. The patient was admitted to the intensive care unit (ICU) on a naloxone drip and was started on intravenous N-acetylcysteine (NAC) at the presumed dose of 150 mg/kg. Shortly after initiating the NAC infusion, the patient developed periorbital edema, skin rash, and hypotension. The infusion of N-acetylcysteine was immediately stopped and the patient required emergent intubation. Resuscitation was begun with intravenous fluids followed by the initiation of phenylephrine. He developed ST elevation in the inferior leads on his ECG. This evolved into an inferior myocardial infarction by ECG and cardiac enzymes. Echocardiogram showed global, severe hypokinesis with an ejection fraction of less than 20% in a patient with no pre-existing cardiac history. Despite aggressive support, he died approximately 17 hours after the initiation of intravenous NAC. Further investigation found a 10-fold formulation error in his NAC loading dose.

**Conclusion:**

The intravenous formulation of NAC has a higher probability of significant adverse effects and complications not described with the oral formulation. Clinicians should be aware of these potential complications when deciding on which formulation to administer.

## Introduction

Acetaminophen is one of the most commonly used over-the-counter (OTC) analgesics and one of the most common causes of poisoning worldwide. Additionally, it is used in combination with both prescription narcotics and OTC medications, making it one of the most accessible medications for potential ingestions. Annually, poison centers receive more than 93,000 calls regarding acetaminophen exposures [1]. Fortunately, antidotal therapy is available for acetaminophen toxicity in the form of N-acetylcysteine (NAC).

The benefits and efficacy of early administration of NAC for acetaminophen toxicity are well described [2]. NAC is thought to have four basic mechanisms of action to limit acetaminophen toxicity: It replenishes endogenous glutathione, directly converts the acetaminophen metabolite N-acetyl-p-benzoquinonimine (NAPQI) to a non-toxic metabolite, facilitates increased formation of N-acetyl-p-aminophenol-sulfate, and buffers cellular damage from NAPQI by acting as a reducing agent [2]. Regardless of the route of administration, there has not been a reported treatment failure for an acute acetaminophen only ingestion, as predicted by the Rumack-Matthew nomogram, when NAC was given within 8 h of ingestion.

Prior to 2004, the only Food and Drug Administration (FDA) approved formulation available in the US was the oral form, despite widespread use of the intravenous formulation in Europe, Australia, and Canada [3]. With the introduction of the approved intravenous formulation, adverse reactions have become an increasing concern with a well-described anaphylactoid reaction leading to a delay in administration or other more serious complications. In this report, we present a case with an anaphylactoid reaction immediately after intravenous administration of a ten-fold formulation error of NAC associated with a massive myocardial ischemia and death.

### Case report

A 53-year-old male with chronic obstructive pulmonary disease, hepatitis C, alcohol abuse, and chronic back pain presented with altered mental status after a presumed suicide attempt after estrangement from his girlfriend. The patient did not have any known cardiovascular history. Family became concerned after the patient failed to wake up, and 911 was called. The patient was found to be stuporous with pinpoint pupils. An intravenous line was established, and after administration of naloxone the patient became belligerent. After discussion with family and review of his medications, it was determined that the patient likely overdosed on an acetaminophen/hydrocodone product and carisoprodol. He was admitted to the ICU requiring a naloxone drip (0.5 mg/h). He had an acetaminophen level of 49 mcg/ml with an unknown time of ingestion. His transaminase levels and coagulation factors were within normal limits (AST 32 IU/l, ALT 47, and INR 0.97). Because of the detectable acetaminophen level with an unknown time on ingestion, intravenous NAC at 150 mg/kg (84 kg-12,600 mg NAC) was ordered.

**Figure 1:**
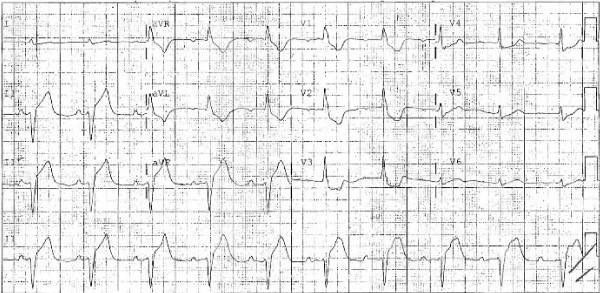
Initial ECG showing pronounced ST segment elevation in the inferior leads

Immediately after initiation of the intravenous NAC, which was to be infused over 1 h, the patient developed periorbital edema, rash, and hypotension with a systolic blood pressure of 80 mmHg. The infusion was stopped. He was rapidly intubated without medications, and resuscitation was initiated with intravenous normal saline. Hypotension persisted despite fluid resuscitation with 2 l normal saline, and therefore phenylephrine (0.1 mg/min) was started. The ECG obtained prior to the administration of phenylepherine showed pronounced ST segment elevation (Figure 1) in the inferior leads, and an emergent cardiology consult was obtained. Because the patient remained unstable he received aspirin and started on heparin, but no further invasive tests or procedures were obtained at that time. His troponin obtained prior to NAC infusion was 0.012 ng/ml and rose to 658 ng/ml over the next 10 h. An echocardiogram obtained 2 h after the initial EKG obtained showed global severe hypokinesis with an ejection fraction of less than 20%. He died approximately 17 h after the initiation of the intravenous NAC loading dose. On further review, a compounding error was discovered that had resulted in an initial dose of 126,000 mg, which was ten-fold greater than the appropriate loading dose for his weight.

## Discussion

This case demonstrates potential difficulties with intravenous NAC as demonstrated by an anaphylactoid reaction. In addition, this case highlights the potential problems of compounding intravenous NAC and demonstrates the dose-dependent nature of the anaphylactoid reaction.

Anaphylactic and anaphylactoid reactions are similar because of the massive release of histamine resulting in hypotension, bronchospasm, pruritus, angioedema, and rash. Anaphylactic reactions are an antibody-mediated mast cell degranulation, which require prior sensitization and production of the IgE antibody. In contrast, anaphylactoid reactions are non-immunogenic and thus do not require previous sensitization. Given the lack of tryptase elevations, non-mast cell sources of histamine likely play an important role in anaphylactoid reactions [4]. It was the recognition of anaphylactoid reactions associated with the intravenous formulation of NAC that precipitated an infusion change in the packet insert, lengthening the infusion time from 15 min to 60 min for the loading dose.

Risk factors for anaphylactoid reactions include atopy, asthma, drug allergy, and low plasma acetaminophen levels, with our patient having the latter [5]. Asthma is one of the better-described risk factors with previous reports of severe anaphylactoid reactions and respiratory distress following intravenous NAC [6]. As for the cardiac manifestations noted in this report, only two other cases reported ECG changes with intravenous NAC. One involved ST depression and T wave inversion, which temporally resolved with antihistamine administration [7]. The other report documents asystole, which responded to precordial thump and intramuscular epinephrine [8]. The current report is unique in the myocardial ischemia associated with intravenous NAC, especially given his only known risk factor for coronary artery disease was smoking.

Many controversies still exist concerning which formulation is more appropriate. Oral administration is thought to improve drug efficacy given its immediate delivery to the liver and the first-pass effect. However, oral formulations have the disadvantage of potential poor tolerance and adherence given the nausea and vomiting associated with administration [9]. In comparison, intravenous formulations have more serious side effects, greater potential for compounding errors, and have been associated with hyponatremia in the pediatric population [10]. Several severe complications from intravenous NAC have been reported, including several fatalities, one in association with asthma [11]. In addition, adverse drug reactions were associated with compounding errors and higher rates of infusion [3].

NAC medication errors are typically classified into the three major categories of systemic calculation, mixing, and measuring errors. These errors with NAC are well described, with systemic calculation errors occurring in 5% of cases, mixing errors in 9%, and measuring errors in 3% [12]. Reports of errors have been reported to be as high as 33% of patients receiving intravenous NAC, with 18.6% having a delay or interruption in therapy for greater than 1 h [13]. Smaller delays in therapy may cause greater elevation in transaminases, increased length of stay, or more serious sequelae. One patient had a delay in her therapy secondary to an anaphylactoid reaction, which may have contributed to her hepatic failure, as the patient ultimately received an orthotopic liver transplant [14].

In an attempt to decrease NAC medication errors, some advocate for a one-bag method as opposed to a three-bag method. The three-bag method mixes a separate bag for each dose during the FDA-approved intravenous NAC protocol. This requires three different volumes and three different concentrations. The alternative one-bag method reduces the likelihood of compounding errors and also reduces delays in administration because nursing staff is not waiting for the pharmacy to deliver the second and third bags. However, this method does require the nursing staff to change the infusion rates, which does provide another potential source for an infusion error.

Previously, both 15-min and 1-h infusions of the loading dose have been used. Both protocols are equally effective for acetaminophen poisoning, but given the dose-dependent relationship of anaphylactoid reactions, 1-h infusions attempt to limit serious reactions. In addition, some have suggested a ceiling weight of 110 kg for obese patients given the volume of distribution, which also reduces the dose received [15]. In pediatric patients, hyponatremia can complicate the intravenous administration given the free water delivery associated with the treatment protocol [10].

## Conclusion

As serious complications with intravenous NAC continue to develop, the clinician must consider the most appropriate route for administration. To date, studies comparing the efficacy and risks of intravenous versus oral NAC have not been completed. Oral NAC can be given through a nasogastric tube for patients that cannot tolerate the oral formulation, but complications of aspiration need to be considered. There is always a potential complication with any therapy; however, clinicians must weigh the risks and magnitude of complications for each individual patient.

## Competing interests

No funding was obtained for this case report. None of the authors have financial disclosures.

## Authors' contributions

AE and MS are responsible for the initial idea and development of the manuscript. All authors contributed to drafting and preparation of the manuscript. All authors read and approved the final manuscript.

## Waiver

Information presented in this report is protected by federal government waiver for poison control centers.
